# The Effect of Weekend and Holiday Resistance Training Combined With a Low-Fat, High-Protein Diet on Endothelial Function, Hepatic Steatosis, and Overall Systemic Health: A Case Report

**DOI:** 10.7759/cureus.100180

**Published:** 2025-12-27

**Authors:** Takayuki Yamaji, Aya Mizobuchi, Chikara Goto, Tatsuya Maruhashi, Yukihito Higashi

**Affiliations:** 1 Research Institute for Radiation Biology and Medicine, Hiroshima University, Hiroshima, JPN; 2 Department of Rehabilitation, Hiroshima International University, Higashihiroshima, JPN

**Keywords:** dietary nutrition, endothelial function, exercise training, flow-mediated vasodilation, metabolic dysfunction-associated steatotic liver disease (masld), weight reduction

## Abstract

The combination of resistance training five or six times per week, along with a low-fat, high-protein diet, is known to promote weight loss and maintain muscle mass. However, for non-athletes, fully replicating this training and diet regimen is challenging. A 39-year-old overweight male was diagnosed with dyslipidemia, including hypertriglyceridemia and high low-density lipoprotein cholesterol, and metabolic dysfunction-associated steatotic liver disease (MASLD), and had decreased flow-mediated vasodilation (FMD). During the eight-month follow-up period, he engaged in resistance training during weekends and holidays in the early mornings, while adhering to a low-fat, high-protein diet. Throughout the follow-up, he underwent blood tests, abdominal ultrasonography, FMD, and body composition measurements every three months. He achieved weight loss, an increase in FMD, and improvements in blood lipid parameters and MASLD. This case suggests the potential benefits of our weight loss method. However, further clinical trials involving individuals with nonspecialized knowledge are needed to establish its general applicability.

## Introduction

Endothelial dysfunction is an initial step in the progression of atherosclerosis. Measurement of flow-mediated vasodilation (FMD) in the brachial artery is a noninvasive, ultrasound-based method used to assess endothelial function. FMD reflects the ability of blood vessels to dilate in response to increased shear stress [1]. It primarily evaluates nitric oxide-dependent vasodilation in the brachial artery. The brachial artery is scanned longitudinally 5 to 10 cm above the elbow using a high-resolution linear ultrasound transducer. A blood pressure cuff is placed around the forearm and inflated to about 50 mmHg above the systolic blood pressure for five minutes. FMD is automatically calculated as the percentage change in peak vessel diameter from the baseline measurement using the following formula: (Peak diameter - Baseline diameter)/Baseline diameter x 100%. FMD decreases from the initial stage of atherosclerosis. In our previous study, we demonstrated that a reduction in FMD was associated with fasting blood glucose (FBG) levels of ≧95 mg/dL, triglyceride levels of ≧106 mg/dL, and also overweight [2-4]. Vlachopoulos et al. showed that patients with nonalcoholic fatty liver disease exhibited significantly lower FMD than control subjects [5]. However, individuals at this initial stage of atherosclerosis often do not qualify for insurance-covered medical treatments, therefore limiting their access to interventional therapies.

Obesity, particularly the increase in visceral fat, is one of the key mechanisms for dyslipidemia, diabetes mellitus, and fatty liver [6], and weight loss is an established intervention for obesity. Although dietary restriction is one of the most popular approaches to weight loss, relying solely on it without exercise can lead to a greater reduction in muscle mass and increase the risk of weight regain, which might be a risk for further sarcopenia [7]. Athletes engaged in physique and bodybuilding typically adhere to a diet combined with resistance training five or six times per week, with fat intake restricted to 10%-20% of total caloric intake and protein intake at 1.6-2.7 g/kg of body weight, to promote weight loss while maintaining muscle mass before competition [8-10]. Another study showed that minimizing muscle mass loss during weight loss is associated with a lower risk of post-weight reduction regain, and an increase in muscle mass might be expected to result in an increase in basal metabolic rate [11]. However, for non-athletes, particularly working-age individuals, securing sufficient time for training might be difficult because of work, household duties, and childcare. Indeed, the lack of time is one of the most commonly reported barriers to maintaining regular exercise among adults [12]. Therefore, developing a time-efficient strategy that can be performed within a limited schedule is essential. Even for working-age individuals, early mornings on weekends and holidays are likely to be times they can use freely.

Therefore, in this case report, we aimed to explore the feasibility and potential effects of a novel, time-restricted resistance training combined with nutritional therapy on body composition, endothelial function, bright liver, and blood parameters.

## Case presentation

A 39-year-old overweight male was diagnosed with dyslipidemia, including hypertriglyceridemia, high low-density lipoprotein cholesterol (LDL-C) levels, and metabolic dysfunction-associated steatotic liver disease (MASLD), which is characterized by hepatic steatosis associated with metabolic dysfunction and is known to increase the risk of cardiovascular disease, and a reduction in FMD during a medical check-up. At the age of 30, while he was working in a busy environment, he gained about 20 kg and developed MASLD. Although he was asymptomatic, dyslipidemia had been pointed out during the annual health check-up. As a certified specialist and researcher in atherosclerosis, he considered that his health condition might influence patients' trust in his medical advice. Therefore, he focused on weight loss and initiated lifestyle modification to improve his health. He had been diagnosed with hypertriglyceridemia and fatty liver but had not received any treatment. Six months ago, he began resistance training because he felt that he was not getting enough physical activity; however, he did not follow any nutritional therapy.

**Figure 1:**
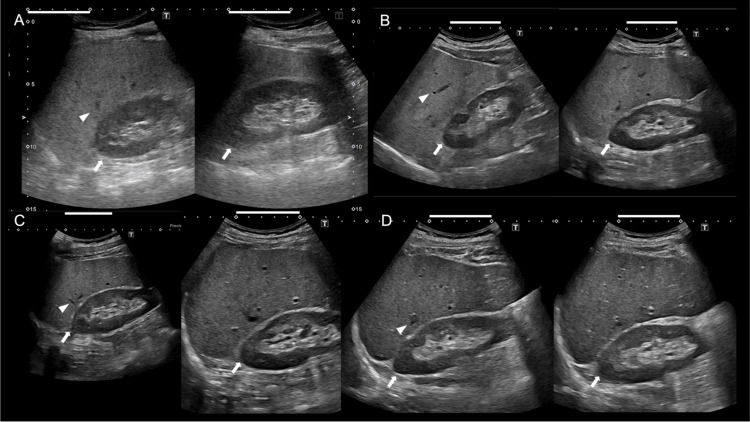
Abdominal echography at baseline (A) and after two (B), five (C), and eight months (D). Arrows indicate the hepatorenal contrast. The contrast appears clear at baseline (A) but becomes progressively less clear at two months (B), five months (C), and eight months (D). Scale bars indicate 5 cm.

The patient fasted the previous night, abstained from drinking alcohol, smoking, and consuming caffeine and vitamins for at least 12 hours before all examinations, including FMD measurement, and refrained from resistance training for 48 hours before all examinations to avoid the acute effects of exercise. All examinations were performed in the supine position in a quiet, dark, temperature-controlled room (23℃ to 26℃). His height, weight, body mass index (BMI), and circumference were 174.5 cm, 77.2 kg, 25.4 kg/m^2^, and 91 cm, respectively. His total cholesterol (TC) level was 210 mg/dL, LDL-C was 140 mg/dL, high-density lipoprotein cholesterol (HDL-C) was 39 mg/dL, triglycerides level was 209 mg/dL, FBG was 97 mg/dL, hemoglobin A1c was 5.5%, aspartate aminotransferase (AST) was 22 IU/L, alanine aminotransferase (ALT) was 47 IU/L, and γ-glutamyl transpeptidase (γ-GTP) was 121 IU/L. His free T3, T4, and thyroid-stimulating hormone (TSH) levels were 3.61 pg/mL, 1.25 ng/dL, and 1.22 μIU/mL, respectively. His FMD level was 4.6%. His InBody analyzer (InBody Japan Inc., Tokyo, Japan) showed a body fat mass of 19.3 kg, a body fat percentage of 25.1%, and soft lean mass of 54.7 kg (Table 1). He was completely abstinent from alcohol (0 g/day). His grandfather had a history of myocardial infarction, and his father had a history of ischemic stroke. Ultrasonography of the abdomen showed a bright liver, hepatorenal echo contrast, and hepatic vascular blurring, while deep attenuation was not observed (Figure 1A). He was categorized as having MASLD based on his BMI, triglycerides, and HDL-C. His FIB-4 index was 0.37, and the MASLD fibrosis score was -4.64. For these clinical conditions, he might have risks of endothelial dysfunction and further cardiovascular events.

**Table 1:** Clinical course during the follow-up period. HDL-C: high-density lipoprotein cholesterol; LDL-C: low-density lipoprotein cholesterol; AST: aspartate aminotransferase; ALT: alanine aminotransferase; γ-GTP: γ-glutamyl transpeptidase; FMD: flow-mediated vasodilation.

	Baseline	After 2 months	After 5 months	After 8 months	Reference ranges
Body weight, kg	77.2	74.5	71.5	69.7	-
Body mass index, kg/m^2^	25.4	24.5	23.0	22.9	18.5-24.9
Waist circumference, cm	91	86	82	80	<90
Total cholesterol, mg/dL	210	170	137	170	150-219
Triglycerides, mg/dL	210	218	126	106	<150
HDL-C, mg/dL	39	30	26	39	40-86
LDL-C, mg/dL	140	95	83	105	70-139
Fasting blood glucose, mg/dL	97	93	92	90	70-109
Hemoglobin A1c, %	5.5	5.5	5.5	5.4	4.6-6.2
AST, IU/L	22	27	21	20	10-40
ALT, IU/L	47	54	35	29	5-40
γ-GTP, IU/L	121	90	73	74	<80
Body fat mass, kg	19.3	16.5	15.3	14.4	-
Body fat percentage, %	25.1	22.1	21.4	20.7	-
Lean mass, kg	57.9	58.0	56.2	55.3	-
Soft lean mass, kg	54.7	54.8	53.1	52.3	-
Skeletal muscle mass, kg	32.9	32.9	31.9	31.3	-
FMD, %	4.6	9.7	10.1	10.3	>7.1

For eight months, he followed a low-fat diet as nutritional therapy and resistance training two or three times per week as exercise therapy. The resistance training was performed using a split training method, on weekends and public holidays in the early mornings, with each training lasting approximately 1.5 hours. In weeks with two training sessions per week, workouts were divided into a day for chest, shoulders, and back, and a day for legs. In weeks with three training sessions per week, workouts were split into a day for legs, a day for chest and shoulder, and a day for arms and back. Training was terminated after 90 minutes, even if some parts remained unfinished.

Chest exercises included barbell bench press or dumbbell bench press, incline Smith press or incline dumbbell press, pec fly or dumbbell fly, and dips. Back exercises included lat pulldown, low row, cable pullover, and chin-up. Shoulder exercises included the Smith machine shoulder press, rear delt fly, and lateral raise. Leg exercises included squat, hip abduction, hip adduction, leg extension, sissy squat, Bulgarian split squat, and leg press or leg curl. Triceps exercises included lying triceps extension, cable press-down, and close-grip bench press or overhead extension. Biceps exercises included incline curl, hammer curl, and preacher dumbbell curl.

Each exercise was performed for three sets of 8-12 repetitions, with rest intervals set at either 1.5 or three minutes.

He did not perform any intentional aerobic exercise. Body weight and body composition were assessed using InBody 470 (InBody Japan Inc.). The basal metabolic rate calculated using InBody was 1620 kcal/day, and that calculated using the Harris-Benedict equation was 1739 kcal/day. His physical activity level was lightly active; therefore, his total daily energy expenditure was 2228 kcal/day, calculated using InBody, and 2391 kcal/day, calculated using the Harris-Benedict equation. On the non-training day, caloric intake was set at 2000-2400 kcal/day, with protein set at 1.6-2 g/kg per day, fat set at 10%-20% of total calories, and the remaining calories were obtained from carbohydrates. In brief, his protein intake was 130-150 g per day, fat at 30-40 g per day, and carbohydrates at 250-300 g per day. On the training day, the caloric intake was set at ≤3000 kcal/day, with protein at 1.6-2 g/kg per day. The nutritional intake consisted of three solid meals and three or four protein drinks per day. Protein was primarily obtained from skinless chicken breast, chicken tenderloin, chicken thigh, or fish. The skin was removed from all meats. Whey protein supplements were consumed three to four times per day. Each serving (30 g) provided 109 kcal of energy, 23.7 g of protein, 0.3 g of fat, and 3.4 g of carbohydrates. Fats were obtained from approximately five unsalted mixed nuts per day, and a small amount of olive oil was used when cooking chicken. Fish was consumed once or twice per week. Carbohydrates were mainly obtained from rice, vegetables, and occasionally pasta or bagels. For pre-workout and post-workout intake, low-fat sweets and low-fat ice cream were consumed. During the training days, protein intake was kept consistent, whereas the remaining dietary intake was adjusted to stay within a total of approximately 3000 kcal. One multivitamin tablet was taken daily as a supplement.

**Figure 2:**
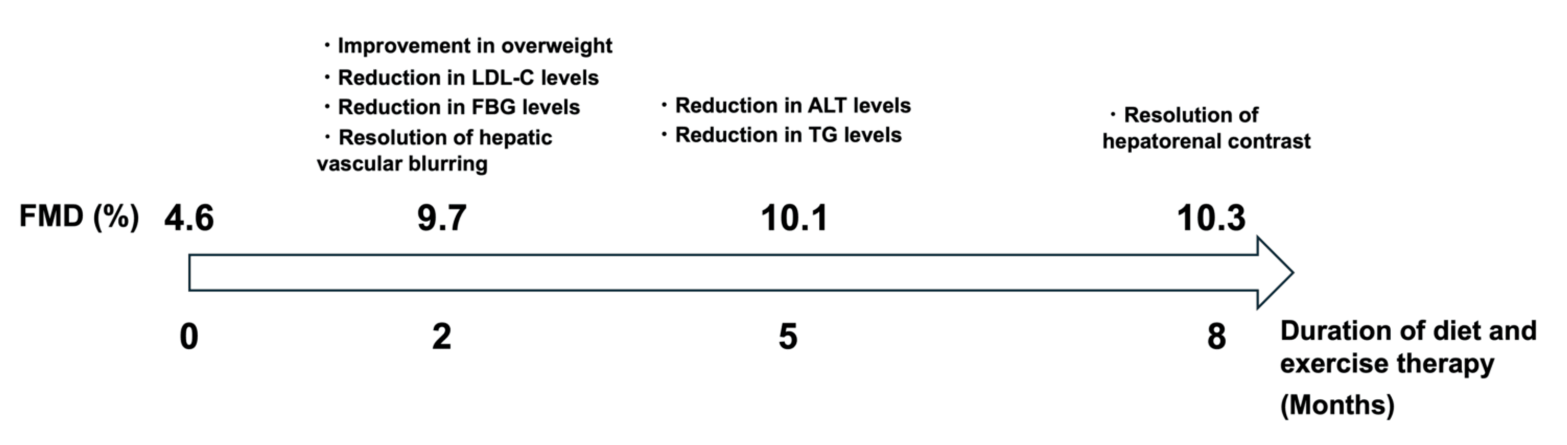
Timeline figure summarizing major milestones. LDL-C: low-density lipoprotein cholesterol; ALT: alanine aminotransferase; FMD: flow-mediated vasodilation; FBG: fasting blood glucose; TG: triglycerides.

Table 1 shows the clinical course during the follow-up period. His body weight decreased from 77.2 to 74.5, 71.5, and 69.7 kg after two, five, and eight months, respectively, resulting in a total weight reduction of 7.5 kg. His waist circumference decreased from 91.0 to 86.0, 82.0, and 80.0 cm after two, five, and eight months, respectively. His BMI decreased from 25.4 to 24.5, 23.0, and 22.9 kg/m^2^ after eight months. His TC level decreased from 210 to 170 mg/dL after two months, 137 mg/dL after five months, and increased to 170 mg/dL after eight months. His triglyceride level increased from 209 to 218 mg/dL after two months, and decreased to 126 and 106 mg/dL after five and eight months, respectively. His HDL-C level decreased from 39 to 30 and 26 mg/dL after two and five months, respectively, and returned to 39 mg/dL after eight months. His ALT level increased from 47 to 54 IU/L after two months, and decreased to 35 and 29 IU/L after five and eight months, respectively. His FMD level increased from 4.6% to 9.7%, 10.1%, and 10.3% after two, five, and eight months, respectively. His body fat mass decreased from 19.3 to 16.5, 15.3, and 14.4 kg after two, five, and eight months, respectively. The body fat percentage decreased from 25.5% to 22.1%, 21.4%, and 20.7% after two, five, and eight months, respectively. The lean mass increased from 57.9 to 58.0 kg after two months and decreased to 56.2 and 55.3 kg after five and eight months, respectively. The soft lean mass increased from 54.7 to 54.8 kg after two months and decreased to 53.1 and 52.3 kg after five and eight months, respectively. The skeletal muscle was similarly 32.9 kg at baseline and after two months, and decreased to 31.9 and 31.3 kg after five and eight months, respectively. Abdominal echography showed improvement in hepatic vascular blurring after two months and improvement in bright liver, while hepatorenal echo contrast remained after five months. At eight months, the hepatorenal echo contrast had disappeared (Figures 1, 2).

## Discussion

In this case report, we presented a patient who achieved a 9.7% reduction in body weight (77.2 to 69.7 kg), a 12.1% reduction in waist circumference (91 to 80 cm), a 49.3% reduction in triglyceride levels (210 to 106 mg/dL), a 25% reduction in LDL-C levels (140 to 105 mg/dL), a 38.3% reduction in ALT levels (47 to 29 IU/L), regression of MASLD, and a 124% improvement in FMD (4.6% to 10.3%) through a combination of resistance training on weekends and holidays in the mornings, and a low-fat, high-protein diet during an eight-month follow-up period. The strength of this diet method is that it is feasible for many people, such as those of working age.

In our case, a decrease in LDL-C and FBG, and an increase in FMD levels were observed after two months, and a decrease in triglyceride levels required five months, and ALT level and fatty liver required eight months. This result indicates that decreased LDL-C and FBG levels might be associated with a reduction in total daily intake, whereas the increased triglycerides and ALT levels and bright liver might be associated with a decrease in body fat. Despite the lack of data from Japanese populations, previous studies conducted outside Japan have suggested that improvement of fatty liver can be expected with a 7%-10% reduction in body weight [13]. InBody data suggest that improvement in endothelial function might be associated with a reduction in body fat mass rather than an increase in muscle mass. A previous meta-analysis showed that resistance training alone does not increase FMD level in patients who were overweight, and our previous study also showed that 12-week high-intensity exercise did not improve endothelial function due to increased oxidative stress [14]. However, this case presents the possibility that the combination of resistance training and nutritional therapy might enable appropriate weight reduction and improvement in endothelial function.

This case report has several limitations. First, the fundamental limitation of reporting a single case precludes any generalizable conclusions about efficacy or practicality. Second, the exceptional motivation and health literacy of the participant limit the applicability to the general population. Third, which component contributed to the outcome could not be clearly identified. For example, irregular sleep patterns and psychological stress caused by ongoing dietary therapy might have affected vascular function. Fourth, the twice-weekly resistance training with dietary therapy did not result in complete maintenance or an increase in muscle mass. More frequent training might have prevented the muscle mass reduction. Fifth, we could not improve HDL-C levels. The combination of aerobic exercise could have contributed to the improvement in HDL-C levels [13]. Brinton et al. reported that a low-fat and low-cholesterol diet resulted in a 29% decrease in HDL-C levels [15]. Kelley et al. found that resistance training did not significantly increase HDL-C levels [16]. Furthermore, fluctuations in HDL-C and LDL-C levels have been reported. During the latter phase of the follow-up period, the rate of weight loss declined, and minor changes in dietary composition might have contributed to the fluctuation in HDL-C levels. Therefore, the observed rebound in HDL-C should be interpreted as an unexplained phenomenon that warrants further investigation. Sixth, in this case report, baseline body weight was within the overweight range, which may have contributed to the rapid improvement in liver enzymes and MASLD. Finally, dietary and exercise adherence were based on patient self-report without independent verification. Therefore, the feasibility of this dietary restriction may be limited to highly motivated individuals. This weight loss strategy may not apply to individuals who are unable to engage in resistance training. However, considering these limitations, this weight loss strategy is useful as an initial approach to weight loss in the working-age population.

## Conclusions

Even without intentional aerobic exercise, a combination of a low-fat, high-protein diet and resistance training might be associated with changes in LDL-C, triglycerides, MASLD, and endothelial function. Fat mass reduction rather than muscle mass increase might have been associated with improvements in FMD levels. This case indicates that such a time-efficient approach might be feasible for individuals with busy schedules; however, more studies are needed to confirm its applicability.
